# Height and prevalence of hypertension in a middle-aged and older Chinese population

**DOI:** 10.1038/srep39480

**Published:** 2016-12-21

**Authors:** Lulu Song, Lijun Shen, Hui Li, Bingqing Liu, Xiaoxuan Zheng, Yuan Liang, Jing Yuan, Youjie Wang

**Affiliations:** 1 MOE Key Lab of Environment and Health, School of Public Health, Tongji Medical College, Huazhong University of Science and Technology, Wuhan, Hubei, China; 2 Department of Maternal and Child Health, School of Public Health, Tongji Medical College, Huazhong University of Science and Technology, Wuhan, Hubei, China; 3 Department of Social Medicine, School of Public Health, Tongji Medical College, Huazhong University of Science and Technology., Wuhan, Hubei, China.

## Abstract

Evidence from epidemiological studies reported that height was inversely associated with cardiovascular diseases, but the association between height and hypertension was unclear. The purpose of this study was to explore the association between height and blood pressure or prevalence of hypertension in a middle-aged and older Chinese population. A total of 33,197 participants aged 37 to 94 years were recruited from the Dongfeng-Tongji cohort study in Hubei province, China. All participants completed baseline questionnaires, medical examinations and provided blood samples. Hypertension was define as a systolic blood pressure (SBP) over 140 mmHg or/and a diastolic blood pressure (DBP) over 90 mmHg, or current use of antihypertensive medication, or participants with self-reported physician diagnosis of hypertension. Multivariate linear and logistic regression models were used. The prevalence of hypertension was 69.1% for men and 58.0% for women. Pulse pressure (PP) and SBP, but not DBP decreased linearly with increasing height among men and women. Comparing the highest with the shortest quartile of height, the multivariate-adjusted odds ratios were 0.80 (95% confidence interval, 0.71, 0.91) for men and 0.83 (0.74, 0.92) for women. In conclusion, height was associated with reduced SBP, PP and prevalence of hypertension in a middle-aged and older Chinese population.

Hypertension is a major risk factor for cardiovascular and kidney diseases globally and constitutes one of the greatest influences on the burden of diseases both in developing and developed countries^1^,^2^,^3^. An analysis from worldwide data reported that nearly one billion people of the world’s adult population had hypertension in 2000, and the total number will increase to 1.56 billion by 2025^4^. Hypertension is also an increasing threat to China’s public health. A recent report indicated that the prevalence of hypertension was 59.5% in aged 50 years and older Chinese population^5^. Epidemiological studies have established risk factors for hypertension, including obesity, high salt intake, alcohol drinking, cigarette smoking, and sedentary lifestyles, but other factors remain unexplored^1^,^6^,^7^.

Height, an easily collected anthropometric measurement, is determined by multiple factors, including socioeconomic status, nutrition, genetic factors, and other biological and physiological factors during childhood^8^,^9^,^10^,^11^,^12^. However, the effects of height on health outcomes are poorly understood. Several studies have explored the association between height and blood pressure or hypertension, but the results remain inconsistent. A prospective birth cohort study suggested that short height was associated with increased pulse pressure (PP) and systolic blood pressure (SBP) but not diastolic blood pressure (DBP) in middle-aged men and women^13^. A cross-sectional study reported that height was inversely correlated with hypertension in women but not in men^14^, while a study from Nigeria suggested that there was no association between height and hypertension^15^. However, most of these studies were conducted in western countries, the evidence relating height and hypertension in China is lacking.

The aim of the present study was to examine the association between height and the prevalence of hypertension in a middle-aged and older Chinese population after adjustment for potential confounders. We hypothesized that short height in adulthood is associated with an increased prevalence of hypertension.

## Results

### Characteristics of the participants

Among the 33,197 eligible participants (14,670 men and 18,527 women) enrolled in our study, the mean (SD) age was 64.35 (8.38) years. The mean (SD) height for men and women were 166.13 (6.00) cm and 156 (9.07) cm, respectively. The prevalence of hypertension was 69.1% for men, 58.0% for women, and 62.9% for all participants.

Table 1 shows age-adjusted characteristics of the participants according to height quartiles. Taller individuals (as compared with those who with short height) were younger, had a higher level of education, were more frequently to consume meat and poultry, soy products, and fruits, and were more likely to have a lower prevalence of stroke (all *P* for trend <0.05), but they had similar prevalence of coronary heart disease and proportion of current smoker and current alcohol drinker. Taller women but men were more likely to have a lower mean BMI and take regular physical activity (all *P* for trend <0.05). Taller men but women had a higher prevalence of diabetes (*P* for trend <0.001).

### Age-adjusted mean blood pressure according to height quartiles

Table 2 presents age-adjusted mean SBP, DBP and PP according to height quartiles. Mean SBP decreased linearly with increasing height among men and women (*P* for trend <0.001). PP showed the same trends as SBP for height in both men and women (*P* for trend <0.001). *Post hoc* analyses showed that taller individuals had lower SBP and PP than those with short height in both men and women (Q2 *vs* Q1, *P* < 0.001; Q3 *vs* Q1, *P* < 0.001; Q4 *vs* Q1, *P* < 0.001).

### Association between height and blood pressure

Table 3 shows the results from multivariate linear regression analyses, which were used to explore the associations between height and blood pressure components. In the age-adjusted analysis, inverse associations were found between height and SBP and PP among men and women (model 1) (all *P* < 0.001). Additional adjustment for BMI (model 2) did not alter the inverse association between height and SBP and PP in both men and women (all *P* < 0.001). In the fully adjusted model (model 3), a linear change in SBP was found to be −0.163 mmHg (95% confidence intervals (CI), −0.242, −0.084) for men and −0.248 mmHg (95% CI, −0.317, −0.179) for women lower for every centimeter increase in height. Each centimeter increase in height was associated with a reduction of 0.216 mmHg for men and 0.272 mmHg for women in PP. No association was found between height and DBP after adjustment for potential factors among men and women.

### Association between height and hypertension

**Figure 1:**
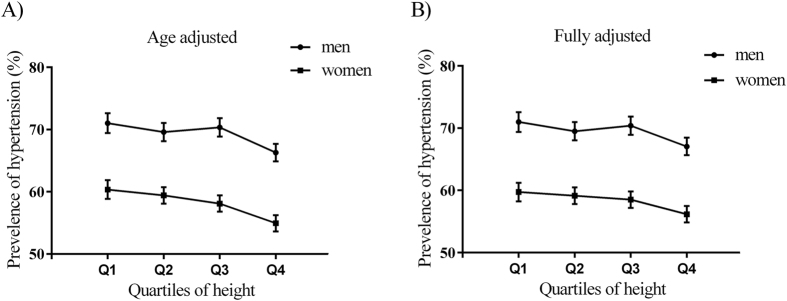
Aged-adjusted and fully adjusted hypertension prevalence by sex across height quartiles in Dongfeng-Tongji cohort study, Shiyan, Hubei, China. (**A**) Association between height and aged-adjusted prevalence of hypertension. (**B**) Association between height and fully adjusted prevalence of hypertension. Fully adjusted confounders included age, body mass index, education, physical activity, marital status, current smoking status, current drinking status, dietary intake frequency categories (including meat and poultry, soy products, vegetables and fruits), osteoporosis, diabetes, stroke, coronary heart disease, and family history of hypertension. The bars represent 95% confidence intervals.

The age-adjusted and fully adjusted prevalence of hypertension according to quartile of height are shown in Fig. 1. For both men and women, there was a descending linear association between height quartile and aged-adjusted prevalence of hypertension. After adjustment for potential confounders, the linear association still remained significant between height and prevalence of hypertension among men and women.

Table 4 displays the age-adjusted and multivariate-adjusted odds ratios (ORs) and 95% CIs for hypertension according to quartiles of height. In the age-adjusted analysis (model 1), increased height was associated with significantly decreased likelihood of hypertension in both men and women (the highest *vs* shortest quartile: OR, 0.80; 95% CI, 0.72, 0.89 for men, OR, 0.78; 95% CI, 0.71, 0.86 for women; *P* for trend <0.001). Further adjustment for BMI (model 2), the inverse association between height and hypertension risk was similarly found with a significant trend (the highest *vs* shortest quartile: OR, 0.79; 95% CI, 0.71, 0.88 for men, OR, 0.86; 95% CI, 0.78, 0.94 for women; *P* for trend <0.001). Adjustment for potential confounders (model 3) did not materially change the association and linear trend (the highest *vs* shortest quartile: OR, 0.80; 95% CI, 0.71, 0.91 for men, OR, 0.83; 95% CI, 0.74, 0.92 for women; *P* for trend <0.001).

### Subgroup analyses

**Figure 2:**
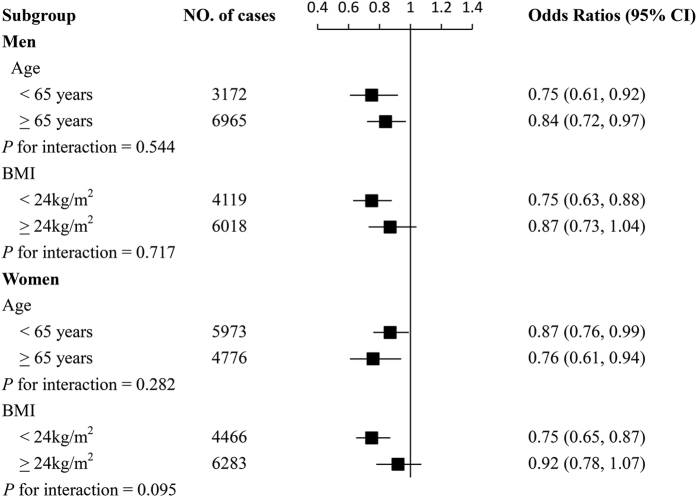
Subgroup analysis of associations between highest quartile of height and hypertension according to age and body mass index. Odds ratios for hypertension are comparison of highest with shortest quartile of height among men and women. Analyses were adjusted for age, body mass index, education, physical activity, marital status, current smoking status, current drinking status, dietary intake frequency categories (including meat and poultry, soy products, vegetables and fruits), osteoporosis, diabetes, stroke, coronary heart disease, and family history of hypertension. Horizontal lines represent 95% confidence intervals.

We further performed stratified analyses according to age and BMI. Figure 2 demonstrated that the inverse associations between height and hypertension were generally similar across subgroup stratified according to age and BMI in both men and women (all *P* for interaction >0.05).

## Discussion

In this cross-sectional study, we found that height was associated with lower SBP and PP but not with DBP independent of potential confounders. We also observed a dose-dependent association between height and prevalence of hypertension after controlling for potential confounders, suggesting that height was associated with a reduced prevalence of hypertension. The inverse association between height and hypertension were similar across subgroup stratified by age and BMI among men and women.

A population-based cohort study of 20,007 individuals aged between 40 and 70 years reported that shorter height in elderly was correlated with an increased risk of hypertension independent of antihypertensive medications; a finding that was in line with our results^16^. In addition, a study from Brazil suggested that short women but not men were associated with prevalence of hypertension after adjustment for age, income, smoking, sodium and alcohol intake and race^17^. A cross-sectional study showed that height was negatively correlated with hypertension in women but not men after controlling for age^14^. These studies supported the finding of the present study that height might be inversely associated with prevalence of hypertension. However, some potential confounders were not fully adjusted in these studies, such as obesity, which might mediate the association between height and hypertension risk. It is well established that obesity is a risk factor for hypertension^18^. Schooling and colleagues demonstrated that short height was not clearly associated with increased blood pressure until adjustment for the cofounding effect of obesity^19^. In the present study, we adjusted for BMI in the multivariate-adjusted regression models, which did not materially change the association between height and hypertension.

Some researchers have explored the association between height and blood pressure components, and the results were inconsistent. A prospective birth cohort study conducted by Langenberg *et al*. reported that shorter height was associated with SBP and PP but not DBP in both middle-aged men and women independent of potential confounders; a finding that was consistent with the present study^13^. Sichieri R *et al*. reported a significant U-shaped association between height and SBP, mainly among women, but no association between height and DBP after adjustment for age, race, income, smoking, salt and alcohol intake, and weight^17^. Potential explanations for the discrepancy may be the differences in racial and ethnic groups under study.

The mechanism underlying the association between height and hypertension is unclear. It is uncertain whether the association is causal or simply mediated by potential factors including socioeconomic status, nutrition in childhood, genetic factors, and other biological and physiological factors during childhood that are important determinants of height^11^,^20^,^21^.

Previous studies found that adverse socioeconomic conditions in childhood were associated with increased SBP and/or DBP in adulthood^22^. Some studies have showed that individuals who grew up in low socioeconomic conditions were more likely to have relatively shorter height, and to have adverse cardiovascular risk factors during adulthood, which were hypothesized to mediate the relationship between height and hypertension^12^,^23^,^24^,^25^,^26^. Adjustment for education, as a marker of socioeconomic conditions in childhood^27^, and other potential confounders did not alter the significant association between height and hypertension, which suggested that the association might not be fully mediated by socioeconomic status and cardiovascular risk factors during adulthood.

The association between height and hypertension may reflect the dynamic properties of the arterial tree. The reflected waves arrive to the central aorta in the late systole or early diastole at a normal pulse wave velocity. Individuals of short stature imply short length of the atrial tree^28^. The shorter length of the atrial tree, the more likely the reflected waves arrive earlier in the central aorta and augment central pressure and PP in late systole^29^. In the present study, we found an inverse association between height and PP, which may be explained by the early reflection waves from a short length of the atrial tree.

Another possible explanation underlying the association between height and hypertension could be nutrition in childhood. Height is not only an important marker of malnutrition in childhood, but also a marker of mothers’ nutrition during pregnancy. Some studies suggested that malnutrition during critical periods in early life might increase the risk of hypertension^30^,^31^. The present study had no data available on nutrition in childhood, which made us not to explore whether height itself or malnutrition have effect on hypertension in adulthood. Further studies are needed to explore the association between height and hypertension independent of nutrition conditions in childhood.

Previous studies suggested that hypertension was a form of accelerated ageing^32^. Given that there was a trend that taller men and women were younger than shorter persons in this study, it is possible that the increased risk of hypertension among shorter persons simply reflects the fact that the older generation may have a greater risk of hypertension than the younger generation. However, we adjusted for age (as a continuous variable) in this study, which did not change the inverse associations between height and hypertension. In addition, we performed a subgroup analysis according to age; the inverse association between height and hypertension were consistent across subgroup stratified by age. Thus the association between height and hypertension was unlikely to have been influenced by age-independent effect of height.

One of the strengths of our study is that it involved a relatively large sample size. In addition, with the use of standard questionnaires and medical measurements, information on socioeconomic status, lifestyles, and medical history of diseases was available and valid, which enhanced the reliability of our results.

There are some limitations that need to be considered. First, as with all previous cross-sectional studies, it is difficult to estimate the causal association between height and hypertension risk. However, it is generally believed that height reaches its peak at young adulthood, and the mean age at diagnosis of hypertension among individuals with self-reported hypertension was 53.2 years in the present study. Second, all participants in the present study were restricted to middle-aged and older population, potentially reducing the generalization of this analysis. Third, height and hypertension might be jointly determined by genetic factors, but we could not estimate their association. Finally, although we had adjusted for some potential confounders, the observed association between height and blood pressure or hypertension might be confounded by other factors that were not measured in our study, such as nutrition in childhood and birth weight.

In conclusion, the results of this study suggested that height, independent of potential confounders, was inversely associated with the prevalence of hypertension in a large middle-aged and older Chinese population. Further studies are needed to identify factors related to the pathogenesis of hypertension to clarify the association between height and hypertension risk.

## Methods

### Study participants

A cross-sectional study design was used in this study. All the participants were selected from the Dongfeng-Tongji cohort study (phase II), which was launched in 2013 in Shiyan City, Hubei Province, China, and conducted by Tongji Medical College, Huazhong University of Science and Technology and Dongfeng Motor Corporation (DMC). Details of the Dongfeng-Tongji cohort study have been described in a former publication^33^. Briefly, a total of 38,295 retired employees of DMC agreed to be enrolled in the Dongfeng-Tongji cohort study in 2013 (phase II). Each participant was required to take a medical examination, provide blood samples, and complete a standard questionnaire by a face-to-face interview. In this study, we excluded participants with either missing value of body height or missing information on diagnosis of hypertension. In total, 5,098 participants were excluded in this study. The final analyses included 33,197 participants (14,670 men and 18,527 women).

The study protocol was approved by the ethics committee of the School of Public Health, Tongji Medical College, Huazhong University of Science and Technology and Dongfeng General Hospital. Written informed consent at enrollment was provided by all participants. All the methods in the present study were carried out in accordance with the approved guidelines.

### Assessment of hypertension

Blood pressure measurements were taken with the participant relaxed and seated straight with the right arm supported in a fixed position. A mercury sphygmomanometer was used to measure SBP and DBP. PP was calculated as the difference between SBP and DBP. In the present study, hypertension was define as a SBP over 140 mmHg or/and a DBP over 90 mmHg, or current use of antihypertensive medication, or participants with self-reported physician diagnosis of hypertension.

### Height

All participants took a medical examination. Height (to the nearest 0.1 cm) was measured in subjects not wearing shoes. Measured height was categorized in sex-specific quartiles (Q). For men, the height quartiles were Q1 < 162 cm, Q2 162–166 cm, Q3 166–170 cm, and Q4 > 170 cm. For women, the corresponding quartiles were Q1 < 152 cm, Q2 152–156 cm, Q3 156–160 cm, and Q4 > 160 cm.

### Assessment of covariates

Information on demographic characteristics (sex, age, marital status, and education), lifestyle factors (current smoking status, current alcohol drinking status, and physical activity), diet (meat and poultry, soy products, vegetables, and fruits), and medical history (hypertension, diabetes, stroke, and coronary heart disease) was obtained from the baseline questionnaire. In our study, current smoking was defined as those who smoked more than 1 cigarette per day in the last 6 months. Current alcohol drinking was defined as those who drank more than once per week in the last 6 months. Physical activity was measured by metabolic equivalent (MET) hours per week. MET hours per week were calculated by the following formula: MET coefficient of activity 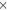 duration (hours per time) 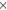 frequency (times per week). For diet, we categorized dietary intake frequency per week into three groups: never, 1–3 times per week, and ≥4 times per week^34^. Items in the medical examination included height, weight, SBP, DBP, bone mineral density (BMD). Osteoporosis was defined as BMD T-score equal to less than 2.5 standard deviations of the average peak BMD. The body mass index (BMI) was calculated by dividing weight in kilograms by height squared in meters.

### Statistical analysis

Categorical variables were described in percentage and compared by Chi-square tests. Continuous variables were presented as mean ± standard deviation (SD) for normally distributed data or median (interquartile range) for skewed data, and compared by one-way analysis of variance (ANOVA) and nonparametric test. *Post hoc* analyses were performed using Dunnett’s test. We used multivariate linear regression models to compare the association of SBP, PP and DBP with height and performed tests for linear trend across height quartiles. Since participants taking antihypertensive medication were more likely to have normal SBP and DBP, they were excluded from the calculation of mean SBP and DBP and the multiple linear regression analyses. Logistic regression models were used to estimated ORs and 95% CIs, which were used to measure the association between height and risk of hypertension after adjustment for potential confounders. We tested for linear trend between height and blood pressure or risk of hypertension and estimated the statistical significance by modeling the median values of quartiles of height as a continuous variable. Multivariate linear and logistic regression models used in our study were adjusted for potential and established risk factors for hypertension: age (continuous); marital status (unmarried or divorced, married); education (elementary or below, junior high school, high school, college or above); current smoking status (yes or no); alcohol drinking status (yes or no); physical activity (continuous); dietary intake frequency categories (including meat and poultry, soy products, vegetables and fruits), body mass index (continuous); prevalent diabetes, stroke and coronary heart disease (yes or no); osteoporosis (yes or no); family history of hypertension (yes or no). Models were fit using height as categorical variables, based on the quartile distribution of height, and the shortest quartile was defined as the referent group. Subgroup analyses were also performed to explore the association between height and hypertension according to age (<65 or ≥65 years) and BMI (<24 or ≥24 kg/m^2^) among men and women. The tests for interaction across subgroup were performed using Wald test. All statistical analyses were performed by using the SAS version 9.4 (SAS institute Inc., Cary, NC). All statistical tests were two-tailed, and the cutoff of significant level was defined as *P* < 0.05.

## Additional Information

**How to cite this article**: Song, L. *et al*. Height and prevalence of hypertension in a middle-aged and older Chinese population. *Sci. Rep.* **6**, 39480; doi: 10.1038/srep39480 (2016).

**Publisher's note:** Springer Nature remains neutral with regard to jurisdictional claims in published maps and institutional affiliations.

## Figures and Tables

**Table 1:** Age-adjusted characteristics of the participants in Dongfeng-Tongji cohort study, according to height quartiles, Shiyan, Hubei, China.

Variable	Height quartile				*P* for trend^†^
	Q1 (lowest)	Q2	Q3	Q4 (highest)	
Men					
Age (years)	69.11 ± 6.65	67.95 ± 6.46	67.61 ± 6.51	66.10 ± 6.90	<0.001
BMI (kg/m^2^)	24.32 ± 3.21	24.34 ± 3.20	24.29 ± 3.20	24.26 ± 3.22	0.296
Married (%)	92.6	92.3	92.6	92.5	<0.901
Education (%)					<0.001
Elementary or below	29.1	24.4	21.8	16.5	
Junior high school	38.4	38.9	36.6	35.0	
High school	20.7	22.5	24.7	28.6	
College or above	11.8	14.2	16.9	19.9	
Physical activity (MET, h/week)	22 (12–42)	24 (12–42)	24 (14–42)	22 (12–42)	0.120
Current smoker (%)	33.8	34.0	33.0	34.7	0.459
Current alcohol drinker (%)	41.0	39.8	42.1	42.4	0.082
Dietary intake, >4 times per week (%)					
Meat and poultry	40.4	43.0	43.6	46.2	<0.001
Soy products	36.9	38.2	38.9	39.6	0.019
Vegetables	97.0	97.2	97.5	96.6	0.313
Fruits	47.4	49.7	53.4	52.2	<0.001
Osteoporosis (%)	13.5	14.6	13.5	16.2	0.004
Diabetes mellitus (%)	12.9	15.1	14.9	15.2	0.015
Stroke (%)	7.9	6.9	6.0	6.0	<0.001
Coronary heart disease (%)	17.2	17.6	18.8	17.9	0.337
Family history of hypertension (%)	20.7	21.4	23.3	25.2	<0.001
Women					
Age (years)	65.63 ± 9.06	62.68 ± 8.54	60.80 ± 8.11	58.67 ± 7.54	<0.001
BMI (kg/m^2^)	24.54 ± 3.53	24.36 ± 3.45	24.20 ± 3.45	23.88 ± 3.51	<0.001
Married (%)	82.9	85.6	85.1	84.3	0.082
Education (%)					<0.001
Elementary or below	26.7	23.2	20.6	16.5	
Junior high school	38.6	38.9	36.1	35.8	
High school	29.5	31.8	34.2	36.5	
College or above	5.2	6.1	9.1	11.2	
Physical activity (MET, h/week)	21 (9–42)	21 (11–42)	21 (11–42)	21 (11–42)	<0.001
Current smoker (%)	2.0	1.8	1.7	2.1	0.767
Current alcohol drinker (%)	11.1	11.4	11.3	11.0	0.816
Dietary intake, >4 times per week (%)					
Meat and poultry	39.4	40.3	43.4	43.9	<0.001
Soy products	37.8	39.2	41.0	39.9	0.032
Vegetables	97.7	97.5	97.4	97.3	0.322
Fruits	64.8	68.9	70.9	73.4	<0.001
Osteoporosis (%)	25.7	24.8	24.9	23.3	0.015
Diabetes mellitus (%)	12.2	13.9	12.4	12.8	0.733
Stroke (%)	3.2	3.1	2.8	2.3	0.014
Coronary heart disease (%)	14.0	15.0	14.2	14.0	0.928
Family history of hypertension (%)	28.3	31.2	33.7	36.4	<0.001

Note: Abbreviation: BMI, body mass index; SD, standard deviation; MET, metabolic equivalent.

Data are mean ± SD for normally distributed data or median (interquartile range) for skewed data, or percentage.

^†^*P* values for trend were performed by assigning the median value of each quartile and treating the variable as continuous in a separate regression model.

**Table 2:** Age-adjusted mean blood pressure according to height quartiles by sex, Shiyan, Hubei, China^‡^.

Blood pressure	Height quartile				*P* for trend^†^
	Q1 (shortest)	Q2	Q3	Q4 (highest)	
Men					
SBP	139.77 ± 21.44	137.69 ± 21.33^*^	137.88 ± 21.32^*^	135.96 ± 21.50^*^	<0.001
DBP	80.64 ± 12.39	80.30 ± 12.33	81.00 ± 12.32	80.49 ± 12.42	0.971
PP	59.12 ± 14.69	57.41 ± 15.59^*^	56.88 ± 14.58^*^	55.30 ± 14.70^*^	<0.001
Women					
SBP	134.45 ± 21.02	132.71 ± 20.62^*^	131.32 ± 20.62^*^	129.46 ± 20.90^*^	<0.001
DBP	76.55 ± 12.02	76.65 ± 11.79	76.47 ± 11.79	76.39 ± 11.95	0.503
PP	57.89 ± 14.85	56.14 ± 14.57^*^	54.89 ± 14.57^*^	53.06 ± 14.76^*^	<0.001

Note: Abbreviation: SBP, systolic blood pressure; DBP, diastolic blood pressure; PP, pulse pressure.

^‡^Individuals taking antihypertensive medication were excluded.

^†^*P* values for trend were performed by assigning the median value of each quartile and treating the variable as continuous in a separate regression model.

^*^P < 0.001 for the comparison with shortest quartile of height, by using ANVOA and Dunnett’s test for post hoc analysis.

**Table 3:** Regression coefficients (95% CIs) of blood pressure components for each centimeter increase in height by sex, Shiyan, Hubei, China^‡^.

	SBP		DBP		PP	
	Coefficient (95% CI)	*P* value	Coefficient (95% CI)	*P* value	Coefficient (95% CI)	*P* value
Men						
Model 1	−0.236 (−0.310, −0.162)	<0.001	0.003 (−0.040, 0.046)	0.894	−0.239 (−0.290, −0.189)	<0.001
Model 2	−0.226 (−0.298, −0.153)	<0.001	0.009 (−0.033, 0.051)	0.405	−0.235 (−0.285, −0.185)	<0.001
Model 3	−0.163 (−0.242, −0.084)	<0.001	0.044 (−0.002, 0.089)	0.066	−0.216 (−0.272, −0.161)	<0.001
Women						
Model 1	−0.335 (−0.400, −0.271)	<0.001	−0.020 (−0.057, 0.018)	0.303	−0.316 (−0.362, −0.270)	<0.001
Model 2	−0.272 (−0.336, −0.208)	<0.001	0.016 (−0.020, 0.053)	0.864	−0.288 (−0.334, −0.243)	<0.001
Model 3	−0.248 (−0.317, −0.179)	<0.001	0.034 (−0.016, 0.063)	0.244	−0.272 (−0.322, −0.222)	<0.001

Note: Abbreviation: SBP, systolic blood pressure; DBP, diastolic blood pressure; PP, pulse pressure; CI, confidence interval.

^‡^Individuals taking antihypertensive medication were excluded.

Model 1: adjusted for age.

Model 2: adjusted for age, body mass index.

Model 3: adjusted for age, body mass index, education, physical activity, marital status, current smoking status, current drinking status, dietary intake frequency categories (including meat and poultry, soy products, vegetables and fruits), osteoporosis, diabetes, stroke, coronary heart disease, and family history of hypertension.

**Table 4:** Age-adjusted and multivariable adjusted ORs (95% CIs) of hypertension according to height quartiles by sex, Dongfeng-Tongji cohort study, Shiyan, Hubei, China.

	Height quartile				*P* for trend^†^
	Q1 (shortest)	Q2	Q3	Q4 (highest)	
Men					
No. of cases	2310	2554	2531	2742	
Model 1	1.00 (reference)	0.93 (0.84, 1.04)	0.97 (0.87, 1.08)	0.80 (0.72, 0.89)	<0.001
Model 2	1.00 (reference)	0.92 (0.83, 1.03)	0.96 (0.86, 1.08)	0.79 (0.71, 0.88)	<0.001
Model 3	1.00 (reference)	0.92 (0.81, 1.04)	0.97 (0.85, 1.10)	0.80 (0.71, 0.91)	<0.001
Women					
No. of cases	2745	2931	2685	2388	
Model 1	1.00 (reference)	0.96 (0.87,1.05)	0.90 (0.82, 0.99)	0.78 (0.71, 0.86)	<0.001
Model 2	1.00 (reference)	0.99 (0.90, 1.09)	0.95 (0.86, 1.05)	0.86 (0.78, 0.94)	<0.001
Model 3	1.00 (reference)	0.97 (0.87, 1.08)	0.94 (0.84, 1.04)	0.83 (0.74, 0.92)	<0.001

Note: Abbreviation: OR, odds ratio; CI, confidence interval.

^†^*P* values for trend were performed by assigning the median value of each quartile and treating the variable as continuous in a separate regression model.

Model 1: adjusted for age.

Model 2: adjusted for age, body mass index.

Model 3: adjusted for age, body mass index, education, physical activity, marital status, current smoking status, current drinking status, dietary intake frequency categories (including meat and poultry, soy products, vegetables and fruits), osteoporosis, diabetes, stroke, coronary heart disease, and family history of hypertension.
